# Molecular profile of vestibular compensation in the medial vestibular nucleus after unilateral labyrinthectomy

**DOI:** 10.1111/jcmm.18532

**Published:** 2024-07-22

**Authors:** Jun Wang, Dan Liu, E. Tian, Yuejin Zhang, Zhaoqi Guo, Jingyu Chen, Jiaqi Guo, Zhanghong Zhou, Shiyu Shi, Yisheng Lu, Sulin Zhang

**Affiliations:** ^1^ Department of Otorhinolaryngology, Union Hospital, Tongji Medical College Huazhong University of Science and Technology Wuhan China; ^2^ Institute of Otorhinolaryngology, Union Hospital, Tongji Medical College Huazhong University of Science and Technology Wuhan Hubei China; ^3^ Department of Otorhinolaryngology The First Affiliated Hospital of Zhengzhou University Zhengzhou China; ^4^ Department of Physiology, School of Basic Medicine Huazhong University of Science and Technology Wuhan China; ^5^ Department of Rehabilitation, Liyuan Hospital of Tongji Medical College Huazhong University of Science and Technology Wuhan China

**Keywords:** lncRNAs, medial vestibular nucleus, unilateral labyrinthectomy, vestibular compensation

## Abstract

Long non‐coding RNAs (lncRNAs) have emerged as crucial regulators in the central nervous system, yet their role in vestibular compensation remains elusive. To address this knowledge gap, we employed unilateral labyrinthectomy (UL) in rats to establish animal models of peripheral vestibular dysfunction. Utilizing ribonucleic acid sequencing (RNA‐seq), we comprehensively analysed the expression profiles of genes dysregulated in the medial vestibular nucleus (MVN) of these rats at distinct time points: 4 h, 4 days, and 14 days post‐UL. Through trans‐target prediction analysis integrating differentially co‐expressed messenger RNAs (mRNAs) and lncRNAs, we constructed lncRNA‐mRNA regulatory networks. Validation of selected mRNAs and lncRNAs was performed using RT‐qPCR. Our RNA‐seq analysis revealed significant aberrant expression of 3054 lncRNAs and 1135 mRNAs compared to control samples. By applying weighted gene co‐expression network analysis (WGCNA), we identified 11 co‐expressed modules encompassing all genes. Notably, within the MEmagenta module, we observed an initial upregulation of differentially expressed genes (DEGs) at 4 h, followed by downregulation at 4‐ and 14‐days post‐UL. Our findings indicated that 3068 lncRNAs positively regulated 1259 DEGs, while 1482 lncRNAs negatively regulated 433 DEGs in the MVN. The RT‐qPCR results corroborated the RNA‐seq data, validating our findings. This study offers novel insights into the lncRNA‐mRNA expression landscape during vestibular compensation, paving the way for further exploration of lncRNA functions in this context.

## 1 INTRODUCTION

Dizziness or vertigo are symptoms of the vestibular balance disorder that affects 15% to over 20% of adults yearly,^1^ and the lifetime prevalence of moderate to severe is approximately 30%,^1^, ^2^ severely limiting daily activities, reducing the quality of life, and leading to falls or other accidents. Patients experiencing acute unilateral vestibular hypofunction or loss can partially ameliorate over time in a process known as ‘vestibular compensation’, promoting the speed and the degree of vestibular compensation will be a legitimate strategy for treating dizziness or vertigo. Vestibular compensation is a complex process involving distributed synaptic, neuronal, and circuit plasticity.^3^ During vestibular compensation, there is a gradual restoration of resting activity in ipsilateral medial vestibular nucleus (MVN) neurons, and a ‘re‐balancing’ of the resting firing rates both sides, approximately in parallel with the behavioural recovery.^4^, ^5^, ^6^

Non‐coding RNAs (ncRNAs) have become a research focus and have newly been demonstrated to play important roles in multiple biological processes.^7^, ^8^, ^9^ These ncRNAs contain small ncRNAs (<200 nt long), long ncRNAs (lncRNAs) (>200 nt long), and circular RNAs (circRNAs).^7^, ^8^, ^9^ More than 170,000 lncRNAs are annotated in the human genome, which play essential roles in brain development, neuron function and maintenance, and neurodegenerative diseases,^9^, ^10^, ^11^ through negatively or positively regulating local protein‐coding gene expression. However, few lncRNAs in the inner ear are identified as related to hearing loss and disruption of balance sense^12^; notably, no literature reported lncRNA function in the vestibular systems.

Our study had several objectives. First, we wished to explore lncRNAs and messenger RNAs (mRNAs) expression patterns during vestibular compensation in rats after UL. Then, weighted gene co‐expression network analysis (WGCNA) and module‐trait relationships were conducted to select important modules. Subsequently, function enrichment analyses of the module were conducted by Gene Ontology (GO). LncRNA‐mRNA networks were also constructed in significant modules. Finally, we verified several differentially expressed mRNAs and lncRNAs by RT‐qPCR. These results may provide a novel reference for vestibular dysfunction prevention and treatment.

## 2 MATERIALS AND METHODS

### 2.1 Animals

Thirty healthy male Sprague–Dawley (SD) rats (specific pathogen‐free, 8–10 weeks old, 180–220 g) were purchased from the Experimental Animal Center, Tongji Medical College, Huazhong University of Science and Technology, Wuhan, China. Rats were raised in separated cages at a standard temperature of 22 ± 2°C and 40 humidity under a 12‐h light/dark cycle condition, with free access to food and water. Rats were housed for 1 week to adapt to the surroundings before the experiments were started. All procedures were approved by the Animal Ethics Committee of Huazhong University of Science and Technology and were conducted in accordance with the National Institutes of Health ‘Guide for the Care and Use of Laboratory Animals’.

### 2.2 Unilateral labyrinthectomy

Experimental rats were anesthetized with sodium pentobarbital (40 mg/kg, intraperitoneal injection) and subjected to unilateral labyrinthectomy (UL) of the right ear as previously described.^3^, ^13^, ^14^, ^15^ The surgical procedure was performed under the operating microscope. Firstly, a 1.5 cm‐long skin incision was made behind the right external acoustic meatus; the cervical muscles, posterior belly of the digastric muscle, the stylohyoid, and their nerves were spared. Special attention was paid to avoid damage to the pterygopalatine artery. Secondly, the ventral wall of the tympanic bulla was carefully opened, and the labyrinth containing the vestibular sensory organs was approached by breaking the promontory. Thirdly, the vestibule was aspirated using a fine plastic suction pipette, destroyed by mechanical ablation, and then rinsed with 100% ethanol. Finally, the space created by the labyrinthectomy was packed with gelfoam, and the skin wound was sutured. After recovering from anaesthesia, animals were housed in individual cages after UL. For the sham operation (SO) group, animals had their right retroauricular skin incised in the same way as the UL. The tympanic bulla was only opened without destroying the tympanic membranous and ossicles after exposure. The experimental animals were created randomly: sham controls, 4 h, 4 and 14 days following UL. The post‐surgery time points were chosen throughout the compensated stage.^16^, ^17^

Animals were excluded from the study if the following symptoms were observed^14^: (1) loss of more than 20% of the pre‐treatment body weight, (2) ulcer of the cornea, which could occur due to an inadvertent lesion of the facial nerve, (3) bleeding from the tympanic cavity and (4) abnormalities in behavioural scoring, for example convulsions, paresis or hemiataxia.

### 2.3 Behavioural assessment

Behavioural symptoms of vestibular deficits consist of three components: spontaneous nystagmus, head roll tilt and postural asymmetry.^6^, ^18^, ^19^ Each component had a maximum score of 10. The assessment of vestibular deficits is detailed in Table 1. Behavioural testing was done by two experienced investigators blinded to the treatment applied to the rats.

**TABLE 1:** Assessment of vestibular deficits.

Component	Method	Scores
Spontaneous nystagmus	Intensity of spontaneous nystagmus was scored with 6–10 points, with 1 point for every 60 beats per minute (bpm). If spontaneous nystagmus was absent at rest, the animal was touched slightly. If this evoked nystagmus, a score of 1–5 points was given, with 1 point for every 60 bpm.	0–10
Head roll tilt	Spontaneous head roll tilt was scored by estimating the angle between the jaw plane and the horizontal; 10 points were given either for a 90‐degree (deg) angle or if the animal rested recumbent on the lesion side or showed barrel‐rolling toward that side. Seven points correlated with a 60‐deg and 5 points with a 45‐deg angle.	0–10
Postural asymmetry	Spontaneous barrel rolling, 10 points; barrel rolling that occurred after a light touch or blowing, 9 points; taking a lying position without any leg support toward the side of the lesion, 8 points; taking a lying position with leg support toward the side of the lesion, 7 points; turning in one direction or taking a lying position using the legs on the lesion side, 6 points; moving by using both legs, 5 points; wandering around while the head was rarely falling on the lesion side, 4 points; wandering around while the head was leaning on the lesion side, 3 points; if the asymmetry was difficult to detect, 2 points were given; if the postural asymmetry was detectable only when the rat was lifted, 1 point was given.	0–10

### 2.4 Specimen collection

After the successful UL model was confirmed by behavioural assessments, the animals were euthanized by sodium pentobarbital overdose (120 mg/kg) after the last day of behavioural testing. Brain samples were promptly isolated from the deeply anesthetized rats and placed on a rat brain matrix immersed in ice‐cold PBS. Coronal sections were performed to extract the brainstem at two specific locations: 2 and 4 mm rostral to the caudal margin of the fourth ventricle. The lateral border of the fourth ventricle and the brown‐coloured boundary between the prepositus hypoglossal nucleus and the dorsal paragigantocellular nucleus were used as reference points for identifying the MVN. A single tissue sample was then extracted from each MVN in the caudo‐rostral direction.^20^, ^21^, ^22^ The MVN tissues were stored in liquid nitrogen and subsequently at −80°C before analysis.

### 2.5 RNA extraction and sequencing

For each sample, 1 μg of total RNA was used for RNA‐seq library preparation. mRNAs were captured by VAHTS mRNA capture Beads (Vazyme, N401). The purified RNA was treated with RQ1 DNase (Promega) to remove DNA before being used for the directional VAHTS Universal V8 RNA‐seq Library Prep Kit for Illumina (NR605). Polyadenylated mRNAs were purified, fragmented and then converted into double‐strand cDNA. Following end repair and A tailing, the DNAs were ligated to Adaptor (N323). After purification of the ligation product and size fractioning to 300–500 bps, the ligated products were amplified purified, quantified and stored at −80°C before sequencing. The strand marked with dUTP (the 2nd cDNA strand) is not amplified, allowing strand‐specific sequencing.

For high‐throughput sequencing, the libraries were prepared following the manufacturer's instructions and applied to Illumina Novaseq 6000 system for 150 nt paired‐end sequencing.

### 2.6 RNA‐seq raw data clean and alignment

Raw reads containing more than 2‐N bases were first discarded. Then adaptors and low‐quality bases were trimmed from raw sequencing reads using FASTX‐Toolkit (Version 0.0.13). The short reads less than 16 nt were also dropped. After that, clean reads were aligned to the mRatBN7.2 genome by HISAT2,^23^ allowing four mismatches. Uniquely mapped reads were used for gene reads number counting and FPKM calculation (fragments per kilobase of transcript per million fragments mapped).^24^

### 2.7 Differentially expressed genes analysis

The R Bioconductor package DESeq2 was utilized to screen out the differentially expressed genes (DEGs).^25^ The fold change >2 or <0.5 and adjusted *p* value < 0.05 were set as the cut‐off criteria for identifying DEGs.

### 2.8 New transcripts predict assembly

Group the RNA‐seq data, use stringtie to assemble the data of each group and predict the transcripts, screen the expression of the predicted transcripts of each group, eliminate the transcripts with FPKM < 1, and then use stringtie^26^ to combine them into one transcript (GTF file).

### 2.9 lncRNA prediction

To predict credible lncRNA, we used four software to predict lncRNA: CPC2,^27^ LGC,^26^ CNCI^28^ and CPAT.^29^ We counted the noncoding transcripts identified by the above four analysis software. After the above steps, we successively removed the transcripts that overlap with the known coding genes, are less than 200 bp in length, have potential coding ability, and are less than 1000 bp away from the nearest gene from the assembly results, obtained the prediction results of new lncRNA, and used the intersection of the four software for subsequent analysis and processing.

### 2.10 Co‐expression network analysis

Set the threshold of co‐location as 100 kb upstream and downstream of lncRNA in the trans‐regulatory relationship pair,^30^ and then calculate the Pearson correlation coefficient between lncRNA and mRNA of co‐location for co‐expression analysis to screen the lncRNA target relationship pairs that meet the absolute value of correlation number greater than 0.6 and *p* value ≤ 0.01, Then take the intersection of the two data sets of co‐location and co‐expression to obtain the cis target of lncRNA.

### 2.11 Functional enrichment analysis

To sort out functional categories of DEGs, Gene Ontology (GO) terms were identified using KOBAS 2.0 server.^31^ The GO function contains three domains: the molecular actions of gene products, the biological processes of those actions and the cellular locations in which they occur.^32^ Hypergeometric test and Benjamini‐Hochberg FDR controlling procedure were used to define the enrichment of each term.

### 2.12 Real‐time qPCR validation

GAPDH (glyceraldehyde‐3‐phosphate dehydrogenase) was used as a control gene for assessing the relative expression of specific genes. cDNA synthesis was done by standard procedures, and RT‐qPCR was performed on the QuantStudio5 with Hieff™ qPCR SYBR® Green Master Mix (Low Rox Plus; YEASEN, China). The concentration of each transcript was then normalized to GAPDH mRNA level using 2^−ΔΔCt^ method.^33^ The primer sequences are listed in Table S1. All the experimental data are presented as the mean ± standard error of the mean (SEM). Statistical analyses were conducted using GraphPad Prism (Version 8.0.2; GraphPad Software Inc, USA). Various data differed from time points and were analysed by repeated measures ANOVA followed by the Bonferroni post‐hoc test. *p* < 0.05 was considered a statistically significant difference.

## 3 RESULTS

### 3.1 Evaluation of the UL rat model

**FIGURE 1:**
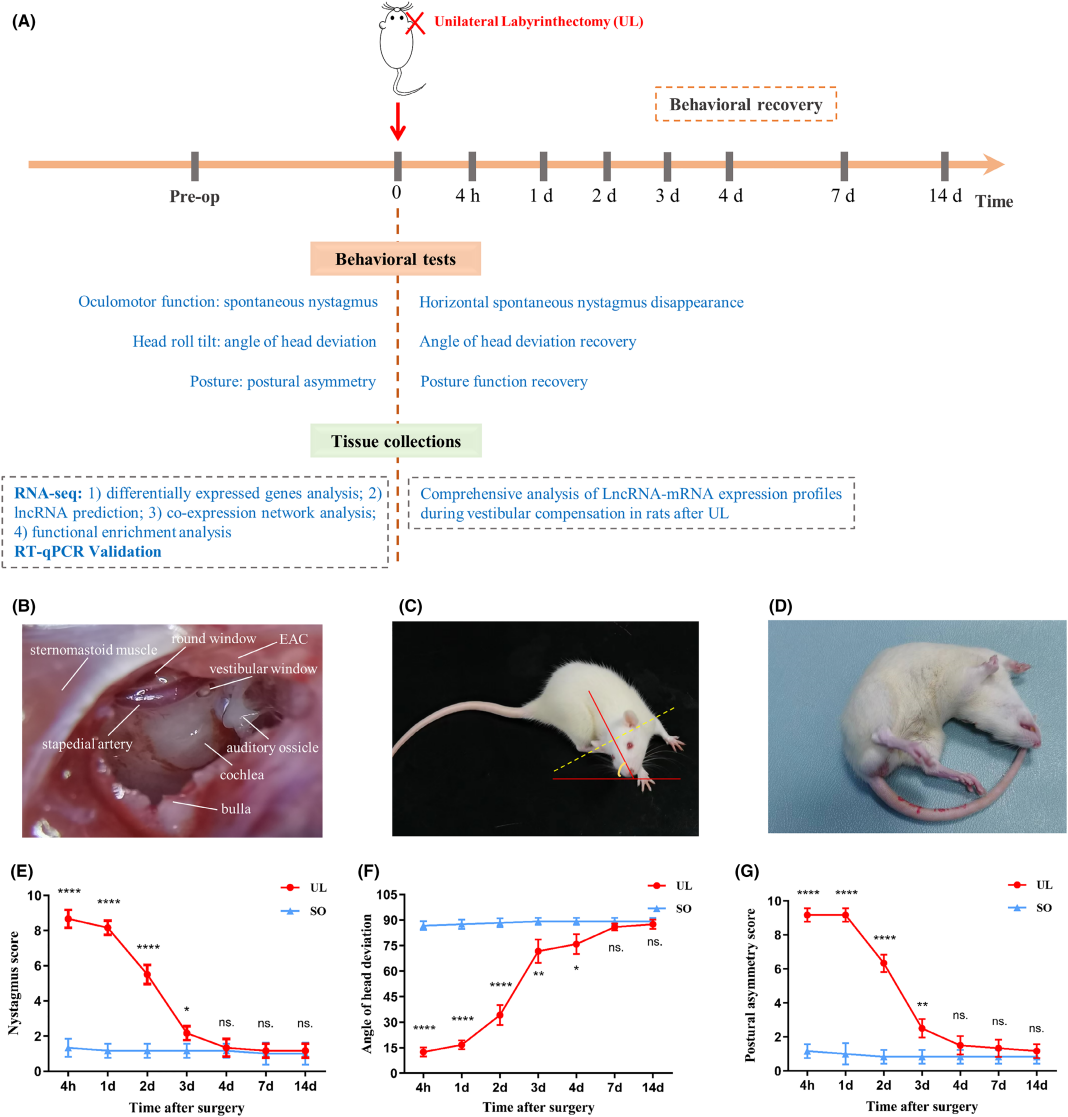
Evaluation of the UL Rat Model. (A) Behavioural investigation of the UL was made at 4 h, 1, 2, 3, 4, 7 days, and 14 post‐lesioned days. MVN tissues were collected for RNA‐seq and RT‐qPCR validation at three representative timepoints (e.g., 4 h, 4 and 14 days). (B–G) UL: Key steps are shown (1) exposing the bulla, (2) opening the bulla and (3) destroying the vestibule. The following anatomical landmarks stand out: sternomastoid muscle, bulla, stapedial artery, round window, vestibular window and EAC (B). After UL, all rats exhibited spontaneous head roll tilt (C, F), postural asymmetry (D, G) and nystagmus (E), and these behaviour phenotypes. Head roll tilt was scored according to the angle between the jaw and the horizontal plane (C). After UL, nystagmus significantly decreased 3 days and disappeared 4 days in almost all of the rats (post hoc test: UL vs. SO, *p* = 0.996). Fastbeating component was always directed towards the contralateral side of the lesion (left side) (*F*(6, 60) = 137.8, *p* < 0.0001) (E). After UL, head roll tilt gradually improved over time (*F*(6, 60) = 259.7, *p* < 0.0001) and reached a minimum value approximately 7 d (post hoc test: UL vs. SO, *p* = 0.125) (F). After UL, postural asymmetry gradually improved over time (*F*(6, 60) = 229.3, *p* < 0.0001) and reached a minimum value approximately 4 days (post hoc test: UL vs. SO, *p* = 0.248) (G). Data shown are means ± SEM (*n* = 6 per group). *p < 0.05, **p < 0.01, ***p < 0.001, ****p < 0.0001, n.s. no significant difference, by two‐way ANOVA followed by Bonferroni's post hoc test. Unilateral labyrinthectomy; EAC, external auditory canal; MVN, medial vestibular nucleus; SEM, standard error of the mean; SO, sham operation.

To evaluate the progression of vestibular compensation following UL, the behavioural scoring of postural asymmetry, head roll tilt, circling movement, and nystagmus were performed at the postoperative time of 4 h, 1, 2, 3, 4, 7 and 14 days (Figure 1A). In the UL group, all the rats showed severe vestibular deficits immediately after recovery from the anaesthesia. Acute unilateral vestibular dysfunction relieved progressively over time, 4 days after UL, no difference was observed in all the behavioural tests between UL and SO groups, however, behavioural performances kept improving till 14 days. As expected, the sham‐operated rats showed no ocular motor or postural deficits. Based on these behavioural results of the vestibular compensation time course, we harvested the ipsilateral MVNs on three timepoints, 4 h, 4 and 14 days (Figure 1B–G).

### 3.2 Differentially expressed mRNA and LncRNA profiles

**FIGURE 2:**
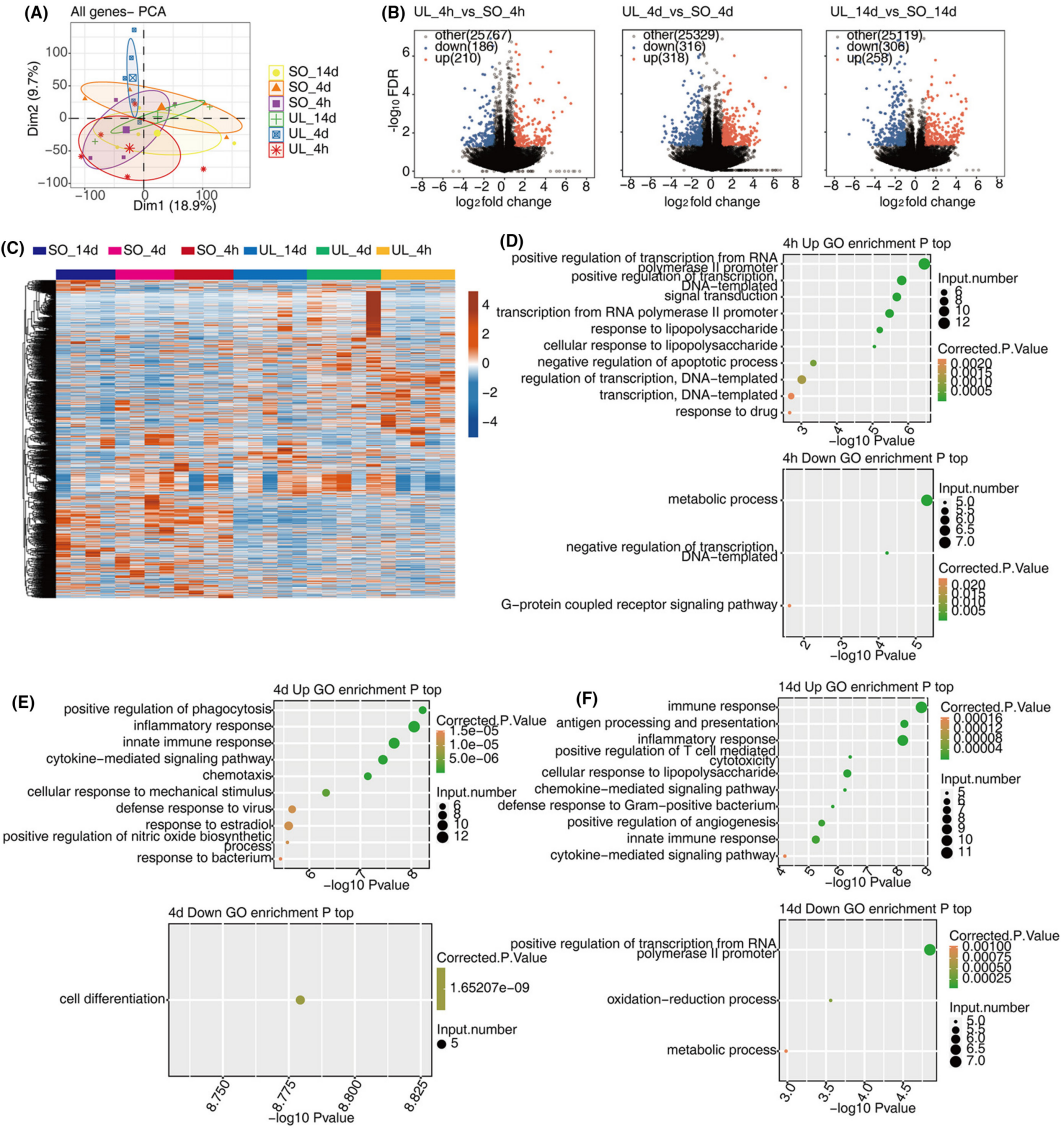
Expression profiles of DEmRNAs during vestibular compensation. (A) PCA based on the expression level of mRNA genes, the samples were clustered by group and recovery time. (B) Volcano plot showing all DEmRNAs between UL and SO groups of three stages. (C) Hierarchical clustering heat map showing expression levels of all DEmRNAs with different colors. (D–F) The Scatter plot exhibiting the most enriched GO biological process results of the dysregulated DEmRNAs in C at different time point. DE, differentially expressed; GO, gene ontology; PCA, principal component analysis; SO, sham operation; UL, unilateral labyrinthectomy.

Principal component analysis (PCA) suggests the expression pattern of mRNA was more separable on 4 days between UL and SO groups, compared to the other two time points, 4 h and 14 days (Figure 2A) though behavioural results could not observe a significant difference between the two groups at 4 days. With the filtering criteria of adjusted *p* value < 0.05 and fold change >2 or <0.5, we identified a total of 1135 dysregulated mRNAs in UL compared to SO groups at three time points (Figure 2B), which could be clustered into groups according to similar gene expression patterns (Figure 2C). Go enrichment analysis shows that genes of the dysregulated mRNAs were enriched in pathways associated with immune response at all the three time points (Figure 2D–F).

**FIGURE 3:**
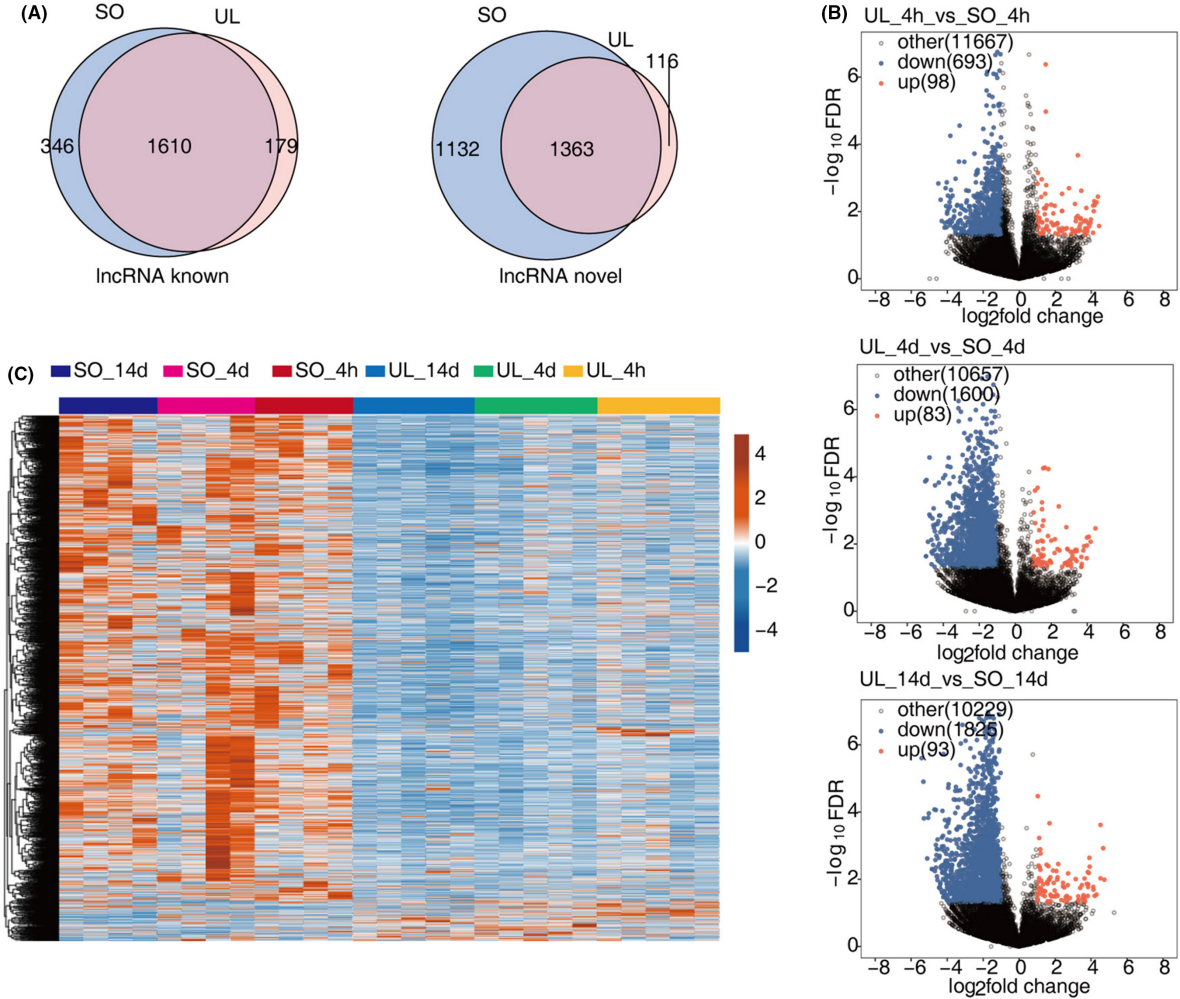
Expression profiles of DElncRNAs during vestibular compensation. (A) Venn diagram of detected known lncRNA (left panel) and novel lncRNA (right panel) in UL and SO groups. (B) Volcano plot showing all DElncRNAs between UL and SO groups of three representative timepoints. (C) Hierarchical clustering heat map showing expression levels of all DElncRNAs. DE, differentially expressed; GO, Gene Ontology; SO, sham operation; UL, unilateral labyrinthectomy.

To explore the global lncRNA expression pattern, we used four software to predict lncRNAs and analysed their expression profiles in UL rats. 3054 significantly dysregulated lncRNAs were identified after filtering the low‐quality and potential protein‐coding transcripts (see Section 2 for detailed information) (Figure 3A). The DElncR results revealed that in contrast to mRNA, most lncRNAs were down‐regulated after UL at three time points (693 down‐regulated and 98 up‐regulated lncRNAs in 4 h; 1600 down‐regulated and 83 up‐regulated lncRNAs in 4 days; 1825 down‐regulated and 93 up‐regulated lncRNAs in 14 days) (Figure 3B,C). These results indicated the regulated expression and potential regulatory roles of lncRNAs in vestibular compensation.

### 3.3 LncRNA‐mRNA co‐expression network analysis

**FIGURE 4:**
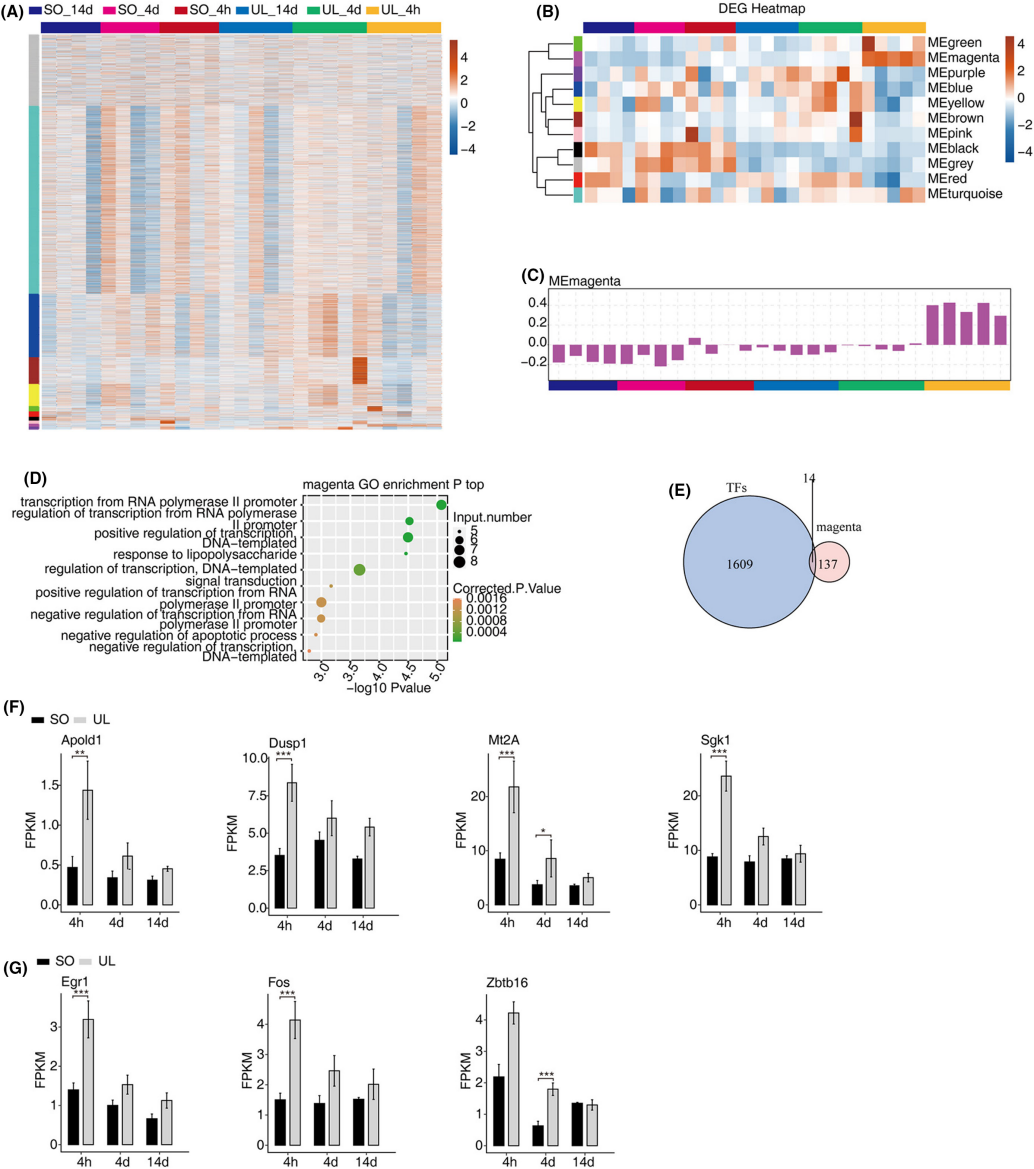
WGCNA analysis of differentially expressed genes in vestibular compensation. (A) Hierarchical clustering heat map showing expression levels of genes detected in all samples by WGCNA. (B) Heat map of module eigen genes sorted by average linkage hierarchical clustering. (C) Magenta bar plot of three stages modules. (D) The Scatter plot exhibiting the most enriched GO biological process results of the magenta modules. (E) Venn diagram showing the overlapped co‐expressed transcription factors (TFs) in MEmagenta module. (F–G) Bar plot showing the expression pattern and statistical difference of some selected DEGs. Data are presented as the mean ± SEM. *p < 0.05, **p < 0.01, ***p < 0.001. DEGs, differentially expressed genes; GO, Gene Ontology; SEM, standard error of mean; SO, sham operation; TFs, transcription factors; UL, unilateral labyrinthectomy; WGCNA, weighted gene co‐expression network analysis.

To further evaluate patterns of expression of the DEGs during vestibular compensation, we performed a weighted gene co‐expression network analysis (WGCNA) (Figure 4A). All DEGs in MVN were divided into 11 co‐expressed modules according to WGCNA results (Figure 4B). In MEmagenta module, the expression of DEGs was upregulated at 4 h and downregulated at 4 and 14 days in the UL group (Figure 4C), and 14 differential expression transcription factors (Figure 4E), which with the highest correlations in vestibular compensation. Therefore, based on the gene ontology (GO) functional analysis (Figure 4D), we sought to select and functionally enrich hub genes (i.e., Apold1, Dusp1, Mt2A, Sgk1, Egr1, Fos and Zbtb16) within the key modules for the FPKM analysis, which might be associated with the progression of vestibular compensation, respectively (Figure 4F,G).

**FIGURE 5:**
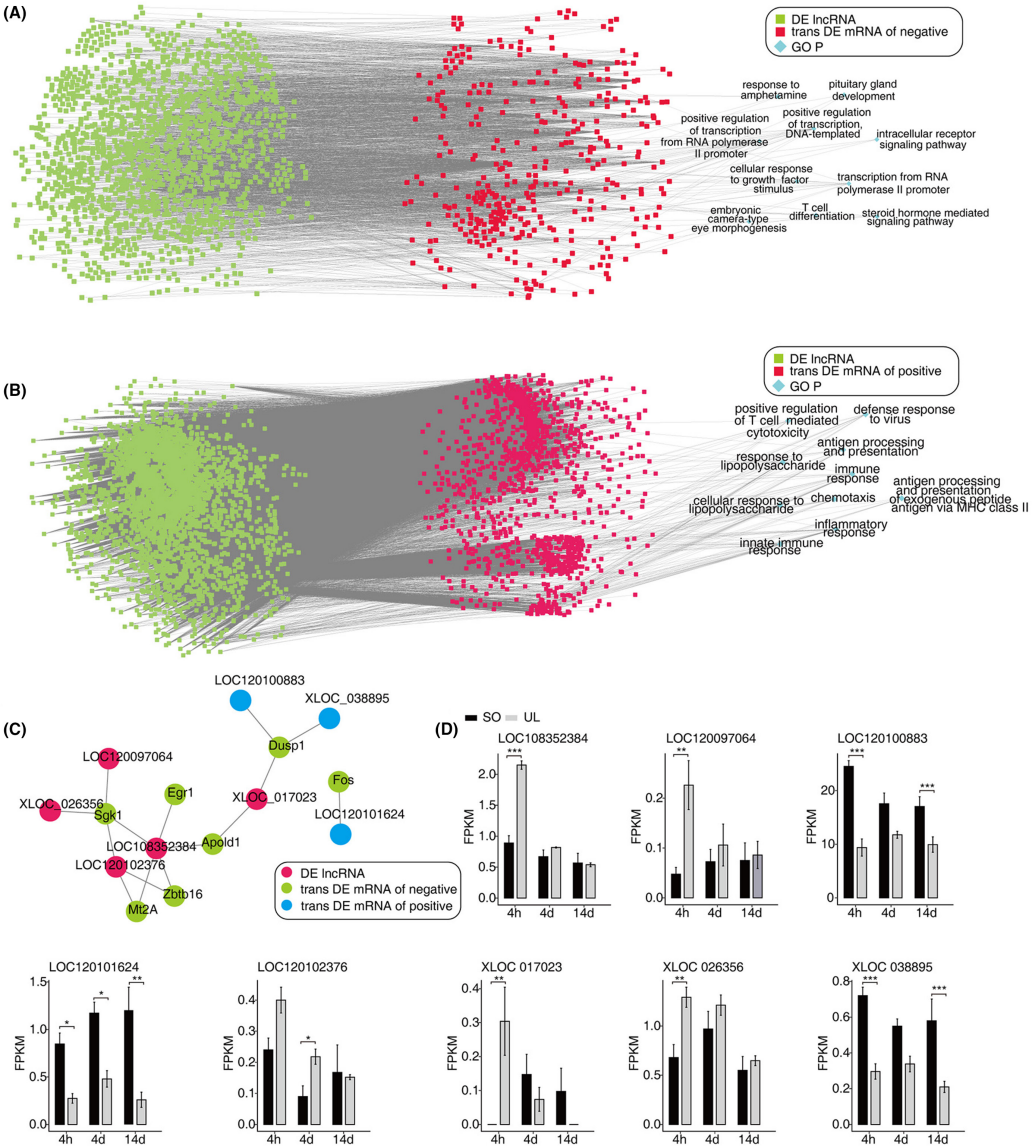
Trans regulatory genes of DElncRNAs associated with vestibular compensation. (A) The top 10 pathways of these trans‐target DEGs that are activated in the vestibular compensation. Cutoffs of *p* value < 0.01 and Pearson coefficient >0.6 were applied to identify the co‐expression pairs. (B) The co‐expressed network between several selected DElncRNAs and DEmRNAs. (C) Bar plot showing the expression pattern and statistical difference of DElncRNAs (D). Data are presented as the mean ± SEM. *p < 0.05, **p < 0.01, ***p < 0.001. DE, differentially expressed; SEM, standard error of mean; SO, sham operation; UL, unilateral labyrinthectomy.

To further explore the potential lncRNAs regulating the aforementioned discrepancy gene, we performed the co‐expression method to construct a network between lncRNAs and mRNAs. We filtered the result by a given threshold, with an absolute correlation coefficient of no less than 0.6 and a *p*‐value less than 0.01. In our study, 3068 lncRNAs positively regulated 1259 DEGs and 1482 lncRNAs negatively regulated 433 DEGs in the MVN (Figure 5A,B). To further reveal the major biological processes of these trans targets DEGs that are activated in the vestibular compensation, we used GO enrichment analysis (Figure 5A,B). The top 10 pathways of these trans‐target DEG of positively enriched biological processes were as follows: (1) antigen processing and presentation, (2) immune response, (3) inflammatory response, (4) innate immune response, (5) chemotaxis, (6) positive regulation of T cell‐mediated cytotoxicity, (7) cellular response to lipopolysaccharide, (8) response to lipopolysaccharide, (9) antigen processing and presentation of exogenous peptide antigen via MHC class II and (10) defence response to the virus.

Finally, we identified eight lncRNAs and their tight association with the above mRNAs to build the sub‐lncRNA‐mRNA networks (Figure 5C,D). These RNA interactions may serve as a novel perspective for exploring the underlying mechanism of vestibular compensation.

### 3.4 Validation of LncRNA‐mRNA expression using RT‐qPCR

**FIGURE 6:**
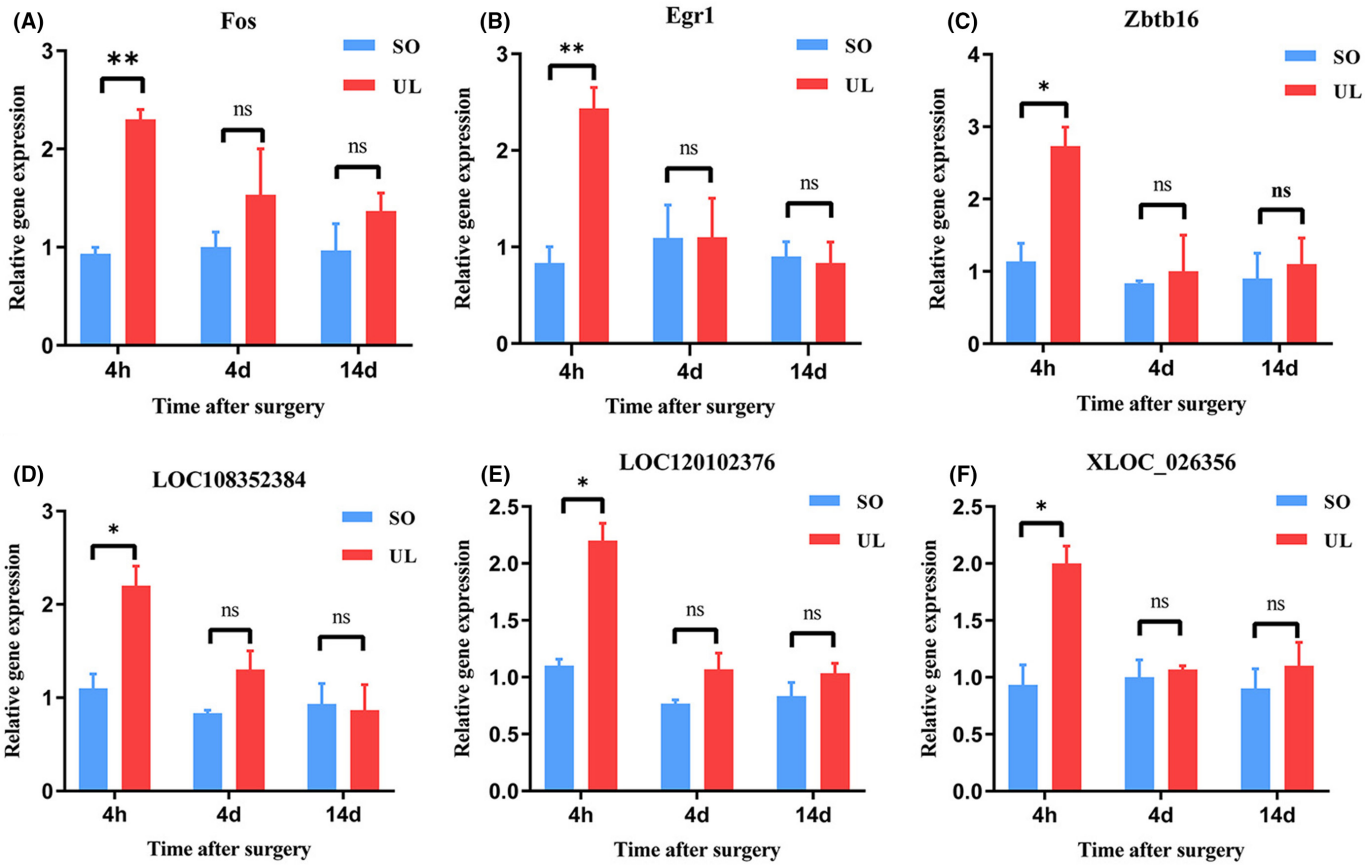
Validation of transcript expression by RT‐qPCR between UL and controls rats. RT‐qPCR was performed for Fos (A), Egr1 (B), Zbtb16 (C), LOC108352384 (D), LOC120102376 (E) and XLOC_026356 (F). Two‐way ANOVA followed by Bonferroni's post hoc test was performed to compare different timepoint between UL and SO groups with significance set at a *p*‐value of <0.05. Data are presented as the mean ± SEM (*n* = 3 per group). *p < 0.05, **p < 0.01, n.s., no significant difference. MVN, medial vestibular nucleus; RTqPCR, the reverse transcription real‐time polymerase chain reaction; SD, Sprague‐Dawley; SEM, standard error of mean; SO, sham operation; UL, unilateral labyrinthectomy.

To test the reliability of the RNA‐seq results, we randomly selected six differentially expressed transcripts: three mRNAs and three lncRNAs in an independent cohort of UL and control rats. As shown in Figure 6, most selected transcripts were detected in control and UL rat MVNs and exhibited significant differential expressions and relatively high verification rates. Generally, RT‐qPCR results confirmed the RNA‐seq data.

## 4 DISCUSSION

In this study, we, for the first time, investigated the altered expression of lncRNAs and mRNAs in MVN tissues comparing UL and control rats. Using microarray analysis, a total of 3054 lncRNAs and 1135 mRNAs were found to be aberrantly expressed. With further bioinformatics analyses including GO analysis and lncRNA‐mRNA co‐expressed network analysis, we studied the potential role of these identified genes and their relationship with the development of vestibular compensation. Our results confirmed the importance of lncRNAs in vestibular dysfunction and further replenished our knowledge about the regulatory roles of lncRNAs during vestibular compensation.

UL has been used as a well‐established and commonly used model for examining vestibular compensation in adult animals.^34^, ^35^ With UL, the deafferented second‐order neurons lose their normally high resting activity, while the contralateral neurons become hyperactive.^34^ In the process of vestibular compensation, different time courses may be divided into ‘static compensation’ and ‘dynamic compensation’.^17^, ^36^, ^37^ In our study, at 4 h after UL represents the acute, uncompensated stage when spontaneous nystagmus is vigorous and postural asymmetry is severe. By the 4th day, these symptoms have diminished significantly, and by the 14th day, the static symptoms have substantially compensated but dynamic reflex deficits remain.^37^ Previous evidence has shown that the recovery of neuronal activity in the ipsilateral MVN after UL, and might play a key role in the compensation process.^13^, ^35^ Therefore, we concentrated this study on only ipsilateral MVN for RNA‐seq data analysis, because they are more relevant to treating symptoms during vestibular compensation.

In previous years, transcriptomic studies focused on analyzing the expression of coding gene transcripts. The mRNA levels of these transcripts were used to study expression patterns that provided clues about the functions of the translated proteins. The rapid evolution of high‐throughput sequencing technologies enabled an unparalleled advance in transcriptomic research, which included discovering and characterizing various new classes of RNA molecules, among them microRNAs, endogenous siRNAs and circRNAs.^38^, ^39^ Another prominent class of RNA molecules discovered was the lncRNAs, which were found to play a critical role in cellular processes such as, but not limited to, differentiation, development and apoptosis.^9^ They regulate the expression of neighbouring protein‐coding genes that have a pivotal role in development and disease progression, and they also regulate gene expression via a transacting mechanism by associating with protein complexes, such as chromatin modifiers, transcription factors, splicing factors, and RNA decay machinery.^7^, ^9^ Most of the identified lncRNAs are still poorly characterized, but most identified mRNAs' functions are well known.^38^, ^40^ To better understand the potential roles of the aberrantly expressed lncRNAs in vestibular compensation, we further conducted WGCNA and GO analysis of the aberrantly expressed mRNAs.

In this study, we identified 3054 lncRNAs and 1135 mRNAs were aberrantly expressed significantly in the UL rats relative to those in control rats. WGCNA has been widely used to reveal the potential expression relationships between protein‐coding genes and lncRNAs. Using WGCNA, we identified that all genes in MVN were divided into 11 co‐expressed modules. In the MEmagenta module, compared with the control group, we found that the expression of DEGs was upregulated at 4 h and downregulated at 4 and 14 days in the process of vestibular compensation. Our results showed that the seven significantly upregulated genes in this module for the identified analysis. Therefore, we concluded that these differentially expressed mRNAs might play crucial roles in the pathogenesis of vestibular compensation.

Given that lncRNAs could negatively or positively regulate local protein‐coding gene expression by inducing chromatin remodelling, transcriptional interference or posttranscriptional regulation, we used the lncRNA‐mRNA network analysis to further explore the potential lncRNAs regulating the aforementioned discrepancy genes. Our results showed that 3068 lncRNAs positively regulated 1259 DEGs and 1482 lncRNAs negatively regulated 433 DEGs in the MVN. Therefore, we speculated that lncRNAs mainly positively regulate DEGs in the acute stage of vestibular compensation.

Using GO analysis, we identified the significant biological functions among the aberrantly expressed protein‐coding genes. As a result, the top 10 pathways of these trans‐target DEGs are positively enriched in inflammatory and immune response pathways. Neuroinflammation is an intricate cellular and molecular process that facilitates the brain's response to various forms of assault, such as injury, infection, or stress.^18^ In line with previous studies, models of acute peripheral vestibulopathies have been reported to induce inflammation and activate reactive plasticity mechanisms.^35^, ^41^, ^42^ Liberge et al.^41^ have indicated that inflammatory factors such as tumour necrosis factor alpha (TNFα) are upregulated in rats following UL at 4 h post‐lesion, while the nuclear factor κB (NFκB) is upregulated at 8 h, peaks at 1 day, and returns to near‐normal values by 3 days; in contrast, manganese superoxide dismutase (Mn‐SOD) shows a slightly delayed upregulation, peaking on the 3rd day and returning to normal levels by the 15th day.^41^ Therefore, we speculate that the relevant inflammatory factors may play an important role in the early stages of vestibular compensation, promoting the recovery of vestibular function by coordinating inflammatory responses and neuroprotective mechanisms.

Furthermore, in this co‐expression network between DE lncRNAs and mRNAs, we found that Sgk1 was correlated with 4 LncRNAs. Sgk1 is a protein kinase that plays a role in various cellular processes, including ion transport, cell survival, proliferation, and apoptosis.^43^, ^44^ Dusp1 was correlated with 3 LncRNAs, and it is considered a stress‐responsive gene and is involved in the cellular response to stress.^45^ The mRNAs Mt2A, Zbtb16 and Apold1 were correlated with 2 LncRNAs, previous study has shown that Mt2A is involved in the anti‐inflammatory response,^46^ Zbtb16 is involved in energy metabolism,^47^ and Apold1 is involved in acute stress response.^48^ In addition, the mRNAs Egr1 and Fos were correlated with one LncRNA. Egr1 can regulate the expression of various genes involved in immune responses, such as cytokines, chemokines, and cell adhesion molecules.^49^ Egr1 is also known to play a critical role in the development and function of the nervous system.^50^ It is expressed in many brain regions, including the hippocampus, cortex, and cerebellum, where it is involved in learning and memory, as well as motor coordination.^50^, ^51^ Fos is a member of the immediate early gene family, which are genes that are rapidly and transiently expressed in response to a wide range of extracellular stimuli, including growth factors, stress, and injury.^52^, ^53^ Therefore, lncRNAs may also be important in MVN by regulating their co‐expressed mRNAs and warrant further research.

Although we identified the aberrantly expressed lncRNAs and mRNAs and investigated their potential roles in the pathophysiology of vestibular compensation, our study suffers from some limitations. Firstly, we used small samples (five biological replicates in the UL group) in our experiments, which may underestimate the number of aberrantly expressed lncRNAs and mRNAs. Secondly, we only predicted the lncRNA functions by identifying the co‐expressed mRNAs and did not verify the exact mechanism involved. Finally, the MVN tissues were collected only in ipsilateral MVN for RNA‐seq data analysis, and contralateral MVN tissues were not studied. Future studies should overcome the above‐mentioned limitations.

## 5 CONCLUSIONS

In conclusion, for the first time, we reported the dysregulated lncRNAs and mRNAs in the MVN from the UL rats. In addition, we predicted the potential functions and regulatory mechanisms of the aberrantly co‐expressed lncRNA‐mRNAs using bioinformatics analyses. We believe that our findings will help to understand the intact regulatory network of lncRNAs in vestibular dysfunction and generate better experimental designs to investigate the lncRNA transcripts in vestibular compensation pathogenesis. Our results may serve as a foundation for further research, which should concentrate on investigating the precise molecular mechanism of these involved genes in the pathophysiological processes of vestibular compensation.

## AUTHOR CONTRIBUTIONS

**Jun Wang:** Formal analysis (supporting); validation (lead); writing – original draft (lead). **Dan Liu:** Methodology (lead); resources (supporting); validation (supporting). **E. Tian:** Formal analysis (equal); visualization (equal). **Yuejin Zhang:** Formal analysis (equal); software (supporting); supervision (supporting). **Zhaoqi Guo:** Supervision (supporting); writing – review and editing (supporting). **Jingyu Chen:** Data curation (supporting); writing – review and editing (supporting). **Jiaqi Guo:** Visualization (supporting); writing – review and editing (supporting). **Zhanghong Zhou:** Visualization (supporting); writing – review and editing (supporting). **Shiyu Shi:** Visualization (supporting); writing – review and editing (supporting). **Yisheng Lu:** Conceptualization (lead); visualization (supporting); writing – review and editing (supporting). **Sulin Zhang:** Conceptualization (equal); funding acquisition (lead).

## FUNDING INFORMATION

This work was supported by grants from the National Key Research and Development Program of China (grant no. 2023YFC2508403), the National Natural Science Foundation of China (grant nos. 82371168 and 82171152), and the Hubei Provincial Key Research and Development Program (grant no. 2023BCB027).

## CONFLICT OF INTEREST STATEMENT

The authors declare that they have no competing interests.

## Supporting information


Table S1

## Data Availability

The sequenced RNA‐seq data in this study are available from the corresponding author on reasonable request.
