# Supplementary material for: Molecular profile of vestibular compensation in the medial vestibular nucleus after unilateral labyrinthectomy
Source: J Cell Mol Med. 2024 Jul 22;28(14):e18532. doi: 10.1111/jcmm.18532 (PMC11263133; doi:10.1111/jcmm.18532)
Supplement: Supplementary file 1 — Table S1 [file JCMM-28-e18532-s001.docx]

**Table S1. Primer sequences used for RT-qPCR.**

| **Gene** | **Forward primer (5’-3’)** | **Reverse Primer (5’-3’)** |
| --- | --- | --- |
| GAPDH | TGCCACTCAGAAGACTGTGG | TTCAGCTCTGGGATGACCTT |
| Fos | CCTGTCTGGTTCCTTCTATG | GGAAGACGTATAGGTAGTGC |
| Egr1 | CCACCTCTTACTCCTCTCC | GGTTGCTGTCATGTCTGAA |
| Zbtb16 | GATAAGGCAGCAGCAAGT | CTTTCTTTCCCAGGGTAGC |
| LOC108352384 | AATCAAGGCAACGGAACA | TAGGCTGCTGTGAAGAGA |
| LOC120102376 | TAAGGCATGGTCCAGACA | TTGTGAAGAGAAGTCAGAGG |
| XLOC_026356 | CCAACCAATAGCAGTGTAC | GAGATTCAGAATAAGCACCT |
